# Dysregulated Peripheral Invariant Natural Killer T Cells in Plaque Psoriasis Patients

**DOI:** 10.3389/fcell.2021.799560

**Published:** 2022-02-03

**Authors:** Yifan Hu, Youdong Chen, Zeyu Chen, Xilin Zhang, ChunYuan Guo, ZengYang Yu, Peng Xu, Lei Sun, Xue Zhou, Yu Gong, Qian Yu, Yuling Shi

**Affiliations:** ^1^ Department of Dermatology, Shanghai Skin Disease Hospital, Tongji University School of Medicine, Shanghai, China; ^2^ Department of Dermatology, Shanghai Tenth People’s Hospital, Tongji University School of Medicine, Shanghai, China; ^3^ Institute of Psoriasis, Tongji University School of Medicine, Shanghai, China

**Keywords:** psoriasis, iNKT cells, IFN-γ, IL-4, IL-17

## Abstract

**Background:** Psoriasis is a common immune-mediated skin disease that involves T-cell-mediated immunity. Invariant natural killer T (*i*NKT) cells are a unique lymphocyte subpopulation that share properties and express surface markers of both NK cells and T cells. Previous reports indicate that *i*NKT cells regulate the development of various inflammatory diseases. IL-17 is a key cytokine in the pathogenesis of psoriasis and a key therapeutic target. Secukinumab is a fully human IgG1κ antibody that targets IL-17A, thereby antagonizing the biological effects of IL-17.

**Objective:** To explore the expression of *i*NKT cells in psoriasis patients and the effect of secukinumab on them.

**Methods:** We examined the frequencies of *i*NKT cells, Tregs, naïve and memory CD4^+^and CD8^+^T cells in the PBMCs as well as their cytokine production in a cohort of 40 patients with moderate-to-severe plaque psoriasis and 40 gender- and age-matched healthy controls. We further collected peripheral blood of another 15 moderate-to-severe plaque psoriasis patients who were treated with secukinumab and evaluated the proportion of *i*NKT cells in the PBMCs at baseline and week 12.

**Results:** The frequencies of conventional CD4^+^ T cells, CD8^+^ T cells, and Tregs in the PBMCs were comparable between psoriasis patients and healthy controls, but the frequencies of Th17 cells, Tc1 cells and Tc17 cells were increased in psoriasis patients. The frequency of peripheral *i*NKT cells and CD69^+^ *i*NKT cells was significantly decreased in psoriasis patients. Both *i*NKT2 cells and *i*NKT17 cells were increased in psoriasis patients, but the ratio of *i*NKT2 cells vs *i*NKT17 cells was significantly reduced in psoriasis patients. After receiving secukinumab, the proportion of *i*NKT cells in the PBMCs of patients was increased, while the proportion of *i*NKT17 cells was decreased.

**Conclusion:** Dysregulated *i*NKT cells may be involved in the pathogenesis of psoriasis and secukinumab may play a regulatory role on *i*NKT cells.

## Introduction

Psoriasis is a common inflammatory skin disease that tends to recur frequently and presently has no cure. The incidence of psoriasis varies worldwide, and its prevalence rate in China is 0.47% (Ding et al., 2012). The development of psoriasis primarily involves T-cell-mediated immunity, and the interleukin (IL)-23/T helper 17 (Th17) cell axis plays an essential role in the pathogenesis of psoriasis (Boehncke and Schon, 2015).

Natural killer T cells (NKT cells) are a unique lymphocyte subpopulation that shares immune properties and expresses surface markers of both natural killer (NK) cells and T cells. Upon activation, NKT cells rapidly produce Th1, Th2, and Th17 cytokines (Brennan et al., 2013). In general, NKT cells are divided into type I (invariant NKT [*i*NKT]) and type II (non-*i*NKT) cells (Godfrey et al., 2004). *i*NKT cells express an invariant TCRα chain consisting of Vα14/Jα18 paired with a limited range of TCRβ chains in mice or Vα24/Jα18 paired with Vβ11 in humans (Koseki et al., 1991; Arase et al., 1992; Lantz and Bendelac, 1994). *i*NKT cells demonstrate CD1d restriction, and *α*-GalCer is the first CD1d-presented lipid antigen for *i*NKT cells (Kawano et al., 1997). Therefore, immunostaining with *α*-GalCer-loaded CD1d tetramers could be useful for precise identification of *i*NKT cells (Kawano et al., 1997).

Based on cytokine production and transcription factor expression, *i*NKT cells can be differentiated into at least three subsets: Th1-like *i*NKT cells (*i*NKT1) that secrete interferon-γ (IFN-γ) and express T-bet (Townsend et al., 2004); Th2-like *i*NKT cells (*i*NKT2) that produce Interleukin-4 (IL-4) and are dependent on the transcription factors PLZF, GATA3, and IRF4 for development (Kim et al., 2006; Lee et al., 2013); and Th17-like *i*NKT cells (iNKT17) that secrete Interleukin-17 (IL-17) and express ROR-γt (Coquet et al., 2008).

*i*NKT cells are a type of key immunoregulatory T cell. *i*NKT cells have been reported to be involved in the development of various inflammatory diseases. They participate in the control of inflammatory bowel disease, allograft tolerance, and regulation of atopic eczema (Seino et al., 2001; Fuss et al., 2004; Simon et al., 2009; Tsuruyama et al., 2012). Previous research showed that the population of NKT cells increases significantly in psoriatic lesions (Bonish et al., 2000; Cameron et al., 2002; Ottaviani et al., 2006; Zhao et al., 2008). In contrast to the accumulation of NKT cells in psoriatic plaques, a few studies have documented decreased proportions and compromised immune activities of NKT cells in the peripheral blood of psoriasis patients (Van Der Vliet et al., 2001; Koreck et al., 2002; Werner et al., 2011). On the contrary, Langewouters *et al.* found an increase in the number of circulating CD94^+^CD161^+^ NKT cells in psoriasis patients (Langewouters et al., 2008). It has been confirmed that human *i*NKT cells can produce IL-17 in a pro-inflammatory environment (Moreira-Teixeira et al., 2011). Mars *et al.* discovered that *i*NKT cells played an important role in limiting the development of the Th17 lineage and provided a natural barrier against Th17 responses in EAE mouse model (Mars et al., 2009). Keunhee’s study also showed that *i*NKT cells can suppress Th17 cell differentiation (Oh et al., 2011). However, the cell surface markers utilized to identify *i*NKT cells in the aforementioned studies, for example, CD3, CD161, and CD94, were not specific to *i*NKT cells. In the present study, we used CD1d tetramers, which are exclusive markers of *i*NKT cells, to accurately identify *i*NKT cells and evaluate their immune functions in psoriasis patients.

Secukinumab is a fully human IgG1κ antibody that targets IL-17A, thereby antagonizing the biological effects of the cytokine. In 2015, secukinumab was approved by the European Medicines Evaluation Agency (EMEA) and the U.S. Food and Drug Administration (FDA) for marketing in Europe and the United States for the treatment of adult moderate-to-severe plaque psoriasis. We also evaluate the proportion of *i*NKT cells in the PBMCs of psoriasis patients treated with secukinumab at baseline and week 12, and analyze whether there is a difference in the proportion of *i*NKT cells before and after treatment.

## Materials and Methods

### Patients

This study was approved by Shanghai Tenth People’s Hospital Ethics Committees (IRB approval number: 2013-RES-14). We collected the peripheral blood of 40 moderate-to-severe plaque psoriasis patients and 40 gender- and age-matched healthy controls from December 2017 to December 2019. The disease severity of psoriasis patients was assessed using the psoriasis area and severity index (PASI) score. Patients’ PASI score were all ≥10 when the blood samples were drawn (Table 1). We also collected peripheral blood of another 15 moderate-to-severe plaque psoriasis patients who were treated with secukinumab before and after the 12 weeks of treatment. Another 15 gender- and age-matched healthy controls’ peripheral blood were also collected. All the participants had no other autoimmune diseases, systemic diseases, malignant tumor or active infections, and had not received systemic therapy for at least 4 weeks or topical therapy for at least 2 weeks. All the procedures were in accordance with the tenets of the Declaration of Helsinki for research involving human subjects. Informed consent was obtained from all the participants, and their clinical information and peripheral blood samples were collected for analysis.

**TABLE 1:** Details of moderate-to-severe plaque psoriasis patients.

Characteristics	Healthy Control (N = 40)	Psoriasis Patients (N = 40)
Age, years, mean (SD)	40.1 ± 10.05	40.85 ± 9.81
Female sex, n (%)	45	45
Body mass index	23.87 ± 2.44	26.34 ± 4.21
Disease duration, years, mean (SD)	-	16.63 ± 9.21
PASI score, mean (SD)	-	18.88 ± 7.63
PGA score, mean (SD)	-	2.25 ± 0.43
BSA (%), mean (SD)	-	39.53 ± 5.59
DLQI score, mean (SD)	-	11.86 ± 7.17

### Treatment and Assessments

15 patients received subcutaneous secukinumab 300 mg at Week 0, 1, 2, 3, 4. After that, they received subcutaneous secukinumab 300 mg every 4 weeks for maintenance treatment. During the treatment, patients should not take any other drugs or physical therapy that may affect the evaluation of efficacy, such as calcineurin inhibitors, glucocorticoids, vitamin D3 derivatives, acitretin, methotrexate, cyclosporine, other biological agents, PUVA and NB-UVB. Patients were recommended to use moisturizing cream daily.

PASI, PGA and BSA score were used for patients’ efficacy assessment.

### Isolation of Peripheral Blood and Flow Cytometry

PBMCs were freshly separated from human peripheral blood using Ficoll-Paque Plus (Catalog# 17-1440-03, GE Healthcare) according to the manufacturer’s recommendations. PBMCs were treated *in vitro* with Cell Stimulation Cocktail (Catalog# 00-4970-03, eBioscience) for 5 h to detect cytokine secretion. To identify dead cells, the cells were first stained with Fixable Viability Stain 780 (Catalog# 565,388, BD Biosciences) for 15 min at room temperature. Subsequently, the cells were stained for 30 min with surface marker antibodies in phosphate-buffered saline containing 2% fetal bovine serum at 4°C. For detecting intracytoplasmic cytokines (IC), the cells were fixed with IC Fixation Buffer (Catalog# 00-8222-49, eBioscience) for 30 min at 4°C. For analyzing intranuclear transcription factors, the cells were fixed with Fixation/Permeabilization Diluent and Concentrate (Catalog# 88-8824-00, eBioscience) at 4 °C for 40 min. After fixation, the cells were stained with intracellular antibodies in Permeabilization Buffer (Catalog# 00-8333-56, eBioscience) at 4 °C for 30 min.

To analyze CD4^+^ T cells, CD8^+^ T cells, regulatory T cells (Treg), and *i*NKT cell frequencies and immunofunctions, PBMCs were stained with the following anti-human antibodies: APC-conjugated *α*-GalCer:CD1d tetramer (NIH tetramer facility, United states), FITC-conjugated anti-CD4 (Catalog# 11-0048-42, eBioscience), APC-conjugated anti-CD4 (Catalog# 17-0049-42, eBioscience), PE-conjugated anti-CD4 (Catalog# 12-0048-42, eBioscience), PE-conjugated anti-CD8 (Catalog# 12-0086-42, eBioscience), PerCP/Cy5.5-conjugated anti-CD8 (Catalog# 301,032, eBioscience), PE-conjugated anti-IL-17A (Catalog# 12-7179-42, eBioscience), APC-conjugated anti-IFN-γ (Catalog# 17-7319-82, eBioscience), PE/cyanine 7 (Cy7)-conjugated anti-IL-4 (Catalog# 25-7049-82, eBioscience), PE-conjugated anti-CD25 (Catalog# 12-0259-42, eBioscience), APC-conjugated anti-forkhead box P3 (FOXP3) (Catalog# 17-4777-42, eBioscience), PE/Cy7-conjugated anti-CD3 (Catalog# 300,420, BioLegend), FITC-conjugated anti-CD69 (Catalog# 11-0699-42, eBioscience), FITC-conjugated anti-CD45RA (Catalog# 11-0458-42, eBioscience), PE/Cy7-conjugated anti-CD45RO (Catalog# 25-0457-42, eBioscience), FITC-conjugated anti-GATA3 (Catalog# 53-9966-42, eBioscience), PE-conjugated anti-ROR-γt (Catalog# 12-6988-82, eBioscience), PerCP/Cy5.5-conjugated anti-T-bet (Catalog# 644,805, Biolegend), and FITC-conjugated anti-IFN-γ (Catalog# 11-7319-82, eBioscience).

Data were acquired on a FACS Canto II (BD Biosciences) and analyzed using the FlowJo software (Tree Star).

### Statistical Analysis

Data are presented as mean ± SD and shown as dot plots of individual samples. Statistical significance was assessed with a two-tailed paired student’s *t*-test. Correlation analysis was performed using the Pearson correlation test. All statistical analyses were performed using the GraphPad Prism software. For all cases, significant differences were considered at *p* values < 0.05.

## Results

### Normal Distribution of Circulating CD4^+^ T Cells, CD8^+^ T Cells and Tregs in Psoriasis Patients

**FIGURE 1:**
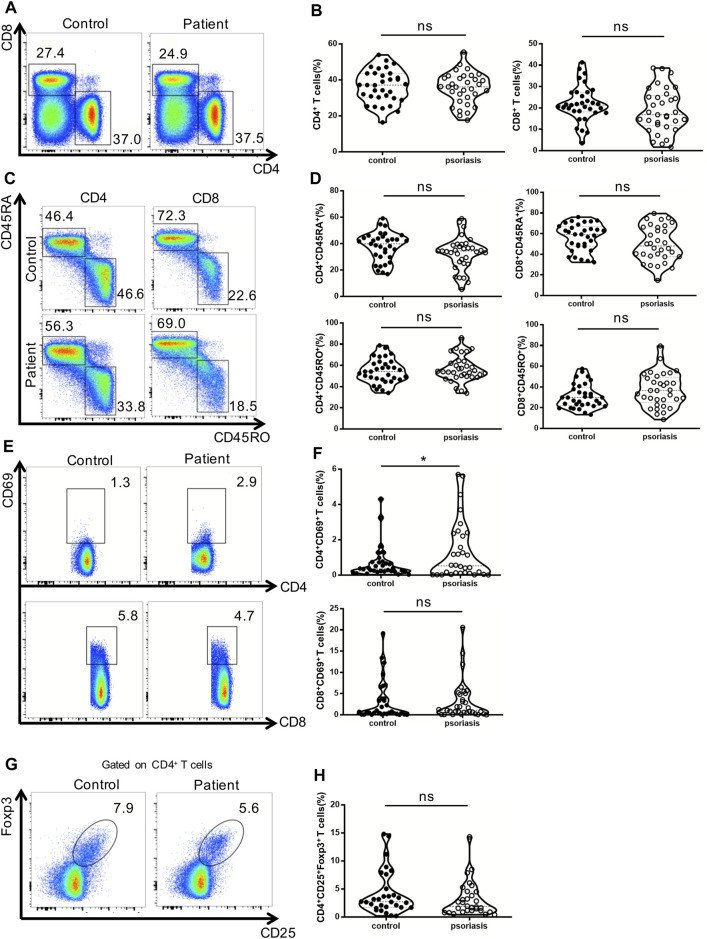
Distribution of conventional T cells and Tregs in the peripheral blood. **(A)** Flow cytometry analysis of CD4^+^ and CD8^+^ T cells in PBMCs from moderate-to-severe plaque psoriasis patients and healthy controls. **(B)** Summary plots showing individual results of the frequency of CD4^+^ and CD8^+^ T cells in moderate-to-severe plaque psoriasis patients versus healthy controls. **(C)** Flow cytometry analysis of memory (CD45RO+) and naïve (CD45RA+) CD4^+^ and CD8^+^ T cells in PBMCs from moderate-to-severe plaque psoriasis patients and healthy controls. **(D)** Summary plots showing individual results of the frequency of CD45RA+ and CD45RO + CD4^+^ and CD8^+^ T cells in moderate-to-severe plaque psoriasis patients versus healthy controls. **(E)** Flow cytometry analysis of CD69 expression in CD4^+^ and CD8^+^ T cells in PBMCs from moderate-to-severe plaque psoriasis patients and healthy controls. **(F)** Summary plots showing individual results of the frequency of CD69 ^+^ CD4^+^ T cells and CD69 ^+^ CD8^+^ T cells in moderate-to-severe plaque psoriasis patients versus healthy controls. **(G)** Flow cytometry analysis of CD4^+^CD25 ^+^ Foxp3+ Tregs in PBMCs from moderate-to-severe plaque psoriasis patients and healthy controls. **(H)** Summary plots showing individual results of the frequency of CD4^+^CD25 ^+^ Foxp3+ Tregs in psoriasis patients versus healthy controls. Data show mean +SEM. *p*-values were determined by paired Student’s t-test. ns, no significance, *p < 0.05, **p < 0.01, ***p < 0.001 and ****p < 0.0001.

First, we examined peripheral conventional T cells by flow cytometry. As shown in Figures 1A, 1B, the percentages of CD4^+^ and CD8^+^ T cells in the PBMCs were comparable between psoriasis patients and healthy controls. There was also no significant difference in the percentages of naïve (CD45RA^+^) and memory (CD45RO^+^) T cells within either the CD4^+^ T cell or CD8^+^ T cell subsets between psoriasis patients and healthy controls (Figures 1C,D). We further analyzed T-cell activation based on the expression of CD69. As shown in Figures 1E,F, a higher number of CD69-positive CD4^+^ T cells were found in the psoriasis patients than in the healthy controls. However, no significant differences were observed in the proportion of CD69-positive CD8^+^ T cells between psoriasis patients and healthy controls. Similarly, no significant difference was detected in the proportion of circulating CD4^+^ CD25^+^ Foxp3^+^ Tregs (Figure 1G,H, gating strategy in Supplementary Figure S1). Thus, our results demonstrated normal distribution of peripheral conventional T cells and overactivation of conventional CD4^+^ T cells.

### Increase in Th17 Cells, Tc1 and Tc17 Cells in the PBMCs of Psoriasis Patients

**FIGURE 2:**
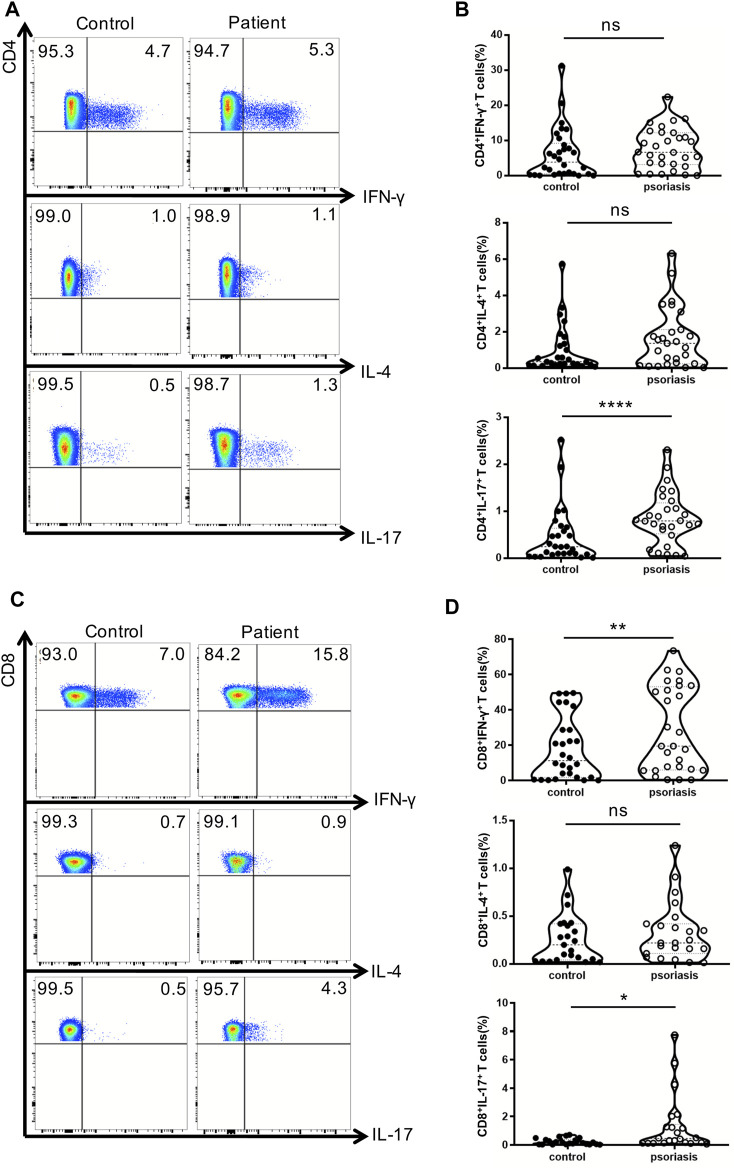
Cytokine-producing T cells from peripheral blood. PBMCs isolated from moderate-to-severe plaque psoriasis patients and healthy controls were stimulated with Cell Stimulation Cocktail for 5 h. The IFN-γ-, IL-4- and IL-17-producing T cells were determined by intracellular staining and flow cytometry analysis. **(A)** The proportion of IFN-γ–,IL-4- and IL-17-producing CD4^+^ T cells in psoriasis patients versus healthy controls. **(B)**Summary plots showing individual results of the frequency of IFN-γ–,IL-4- and IL-17- producing CD4^+^ T cells in psoriasis patients versus healthy controls. **(C)**The proportion of IFN-γ–,IL-4- and IL-17-producing CD8^+^ T cells in psoriasis patients versus healthy controls. **(D)**Summary plots showing individual results of the frequency of IFN-γ-,IL-4- and IL-17- producing CD8^+^ T cells in psoriasis patients versus healthy controls. Data show mean +SEM. *p*-values were determined by paired Student’s t-test. ns, no significance, *p < 0.05, **p < 0.01, ***p < 0.001 and ****p < 0.0001.

Next, we examined the production of IFN-γ, IL-4, and IL-17 by conventional T lymphocytes. There was no significant difference in the percentages of Th1 and Th2 cells between psoriasis patients and healthy individuals (Figures 2A,B). Notably, the proportion of Th17 cells was significantly augmented in the PBMCs of psoriasis patients (Figures 2A,B), as reported in previous studies (Furue et al., 2019). Moreover, the proportions of circulating T cytotoxic 1 (Tc1) and Tc17 cells were significantly upregulated in psoriasis patients (Figures 2C,D).

### Decrease in the Frequency of *i*NKT Cells in the PBMCs of Psoriasis Patients

**FIGURE 3:**
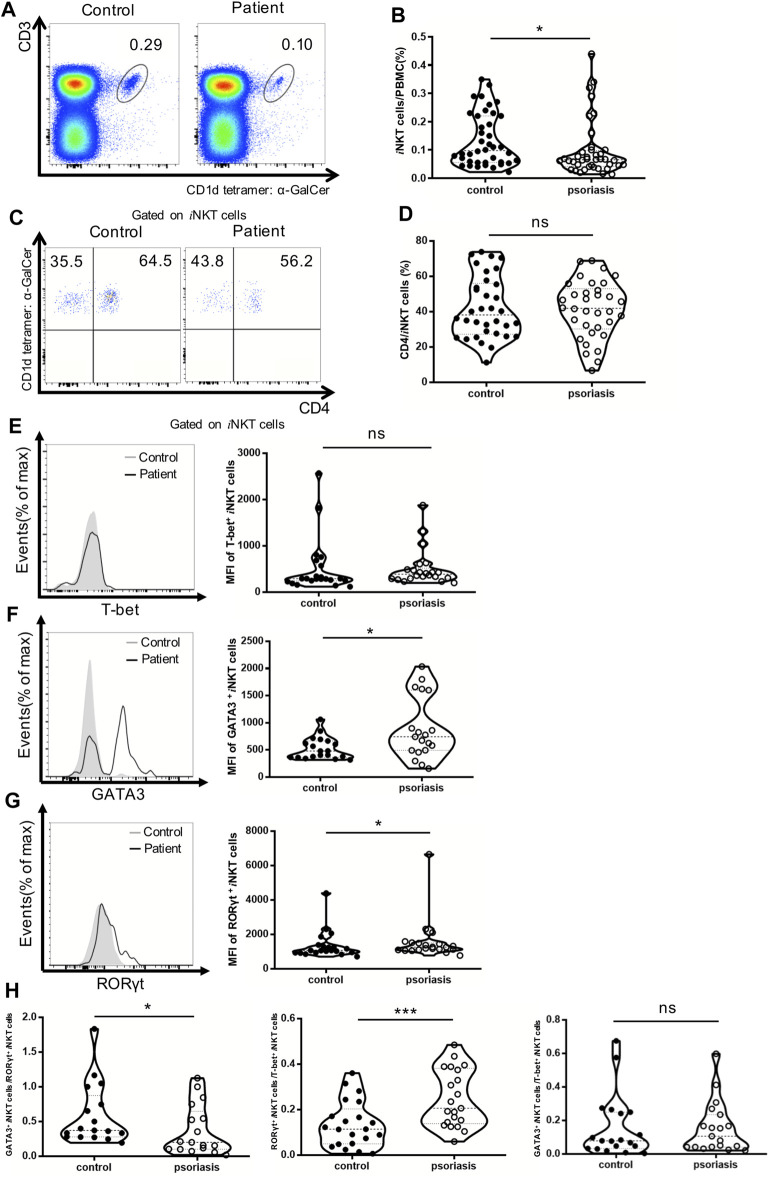
*i*NKT cells frequency and cell subsets in PBMCs of psoriasis patients. **(A)** Representative FACS dot plots for iNKT cells from psoriasis patients and healthy controls. **(B)** Summary plots showing individual results of iNKT cell frequency in psoriasis patients versus healthy controls. **(C)** Representative FACS dot plots for iNKT cell CD4 expression in psoriasis patients and healthy controls. **(D)** Summary plots showing individual results of the frequency of CD4^+^ iNKT cells in psoriasis patients versus healthy controls. **(E)** Representative histogram and summary plots showing individual results of the MFI for T-bet + iNKT cells in psoriasis patients and healthy controls. **(F)** Representative histogram and summary plots showing individual results of the MFI for GATA3+ iNKT cells in psoriasis patients and healthy controls. **(G)** Representative histogram and summary plots showing individual results of the MFI for RORγt + iNKT cells in psoriasis patients and healthy controls. **(H)** Summary plots showing individual results of GATA3+ iNKT cells/RORγt + iNKT cells, RORγt + iNKT cells/T-bet + iNKT cells and GATA3+ iNKT cells//T-bet + iNKT cells in psoriasis patients versus healthy controls. Data show mean +SEM. *p*-values were determined by paired Student’s t-test. ns, no significance, *p < 0.05, **p < 0.01, ***p < 0.001 and ****p < 0.0001.

*i*NKT cells have been shown to play a crucial role in the development of autoimmune diseases (Bendelac et al., 2007). The *α*-GalCer-loaded CD1d tetramer is the best reagent currently available to accurately distinguish *i*NKT cells in terms of specificity and sensitivity (Berzins et al., 2011). As depicted in Figures 3A,B, the proportion of *i*NKT cells in the PBMCs from psoriasis patients was lower than that in healthy controls. This indicates that a defect in *i*NKT cells might be involved in the development of psoriasis. However, there was no correlation between the proportion of *i*NKT cells and PASI score in psoriasis patients (Supplementary Figure S2).

### Increase in *i*NKT2 and *i*NKT17 Cell Sublineages in Psoriasis Patients

Human mature *i*NKT cells can be divided into functionally distinct CD4^+^ and CD4^−^ subsets. CD4^+^ *i*NKT cells produce both Th1 and Th2 cytokines, whereas the CD4^−^ *i*NKT subset mainly exhibits a Th1 cytokine profile (Gumperz et al., 2002). To investigate whether *i*NKT cells from psoriasis patients exhibited phenotypic abnormalities, we first analyzed the proportion of the CD4^+^ *i*NKT subset in the PBMCs, but no significant differences were detected between psoriasis patients and healthy controls (Figures 3C,D).

We further analyzed the sublineages of *i*NKT cells (Figures 3E–G). While the Mean Fluorescence Intensity (MFI) of T-bet^+^ *i*NKT cells (*i*NKT1) remained unaltered, the MFI of GATA3^+^ *i*NKT cells (*i*NKT2) and ROR-γt^+^ *i*NKT cells (*i*NKT17) were significantly increased in psoriasis patients. Moreover, we found that the ratio of GATA3^+^ *i*NKT cells vs ROR-γt^+^ *i*NKT cells decreased and the ratio of ROR-γt^+^ *i*NKT cells vs T-bet^+^ *i*NKT cells increased in psoriasis patients, suggesting that there may be imbalance of *i*NKT cells sublineages in psoriasis (Figure 3H).

### Decrease in *i*NKT Cell Activation and Increased IL-4- and IL-17-Producing *i*NKT Cells in Psoriasis Patients

**FIGURE 4:**
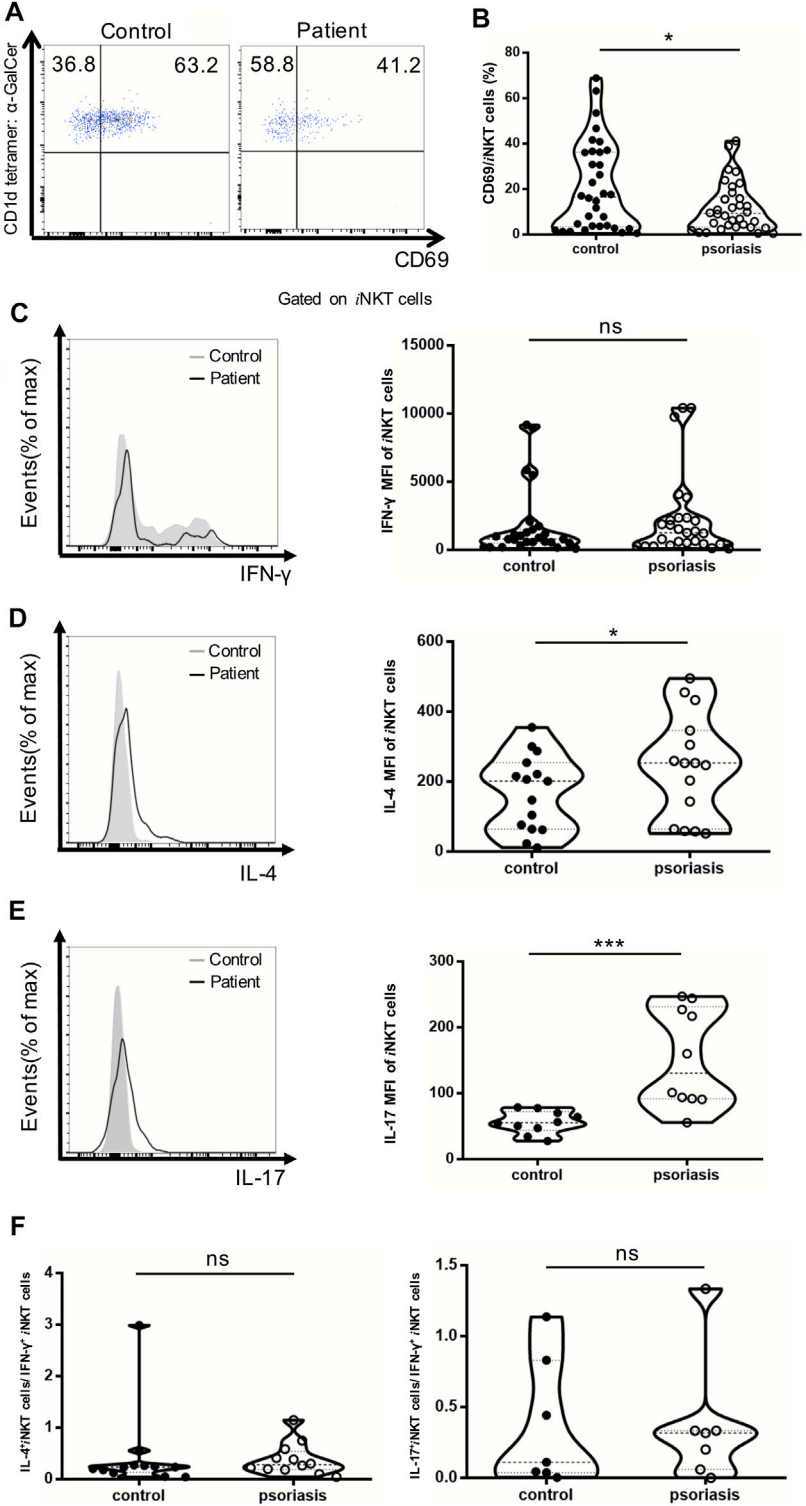
*i*NKT cells activation status and cytokine production in PBMCs of psoriasis patients. Intracellular IFN-γ and IL-4 production of circulating *i*NKT cells was analyzed after stimulation with Cell Stimulation Cocktail for 5 h. **(A)** Representative FACS dot plots for iNKT cell CD69 expression in psoriasis patients and healthy controls. **(B)** Summary plots showing individual results of the frequency of CD69 ^+^ iNKT cells in psoriasis patients versus healthy controls. **(C)** Representative histogram and summary plots showing individual results of the MFI of IFN-γ-producing iNKT cells in psoriasis patients versus healthy controls. **(D)** Representative histogram and summary plots showing individual results of the MFI of IL-4-producing iNKT cells in psoriasis patients versus healthy controls. **(E)** Representative histogram and summary plots showing individual results of the MFI of IL-17-producing iNKT cells in psoriasis patients versus healthy controls. **(F)** Summary plots showing individual results of IL-4-producing *i*NKT cells/IFN-γ-producing *i*NKT cells and IL-17-producing *i*NKT cells/IFN-γ-producing *i*NKT cells in psoriasis patients versus healthy controls. Data show mean +SEM. *p*-values were determined by paired Student’s t-test. ns, no significance, *p < 0.05, **p < 0.01, ***p < 0.001 and ****p < 0.0001.

CD69 has been utilized as a cell-surface marker of *i*NKT cell maturation and activation. The percentage of CD69^+^ *i*NKT cells was reduced in the PBMCs of psoriasis patients, suggesting that *i*NKT cells are less activated in psoriasis patients competed to healthy controls (Figures 4A,B). In addition, the MFI of IL-4- and IL-17-producing *i*NKT cells were significantly increased in psoriasis patients, whereas no significant difference was detected in the MFI of IFN-γ-producing *i*NKT cells between psoriasis patients and healthy controls (Figures 4C–E). This was in accordance with our findings for the *i*NKT cell sublineages. Furthermore, there was no significant difference in the ratio of IL-17-producing *i*NKT cells vs IFN-γ-producing *i*NKT cells and the ratio of IL-4-producing *i*NKT cells vs IFN-γ-producing *i*NKT cells between psoriasis patients and healthy controls (Figure 4F).

### Increase in the Frequency of *i*NKT Cells in Psoriasis Patients After Receiving Secukinumab

**FIGURE 5:**
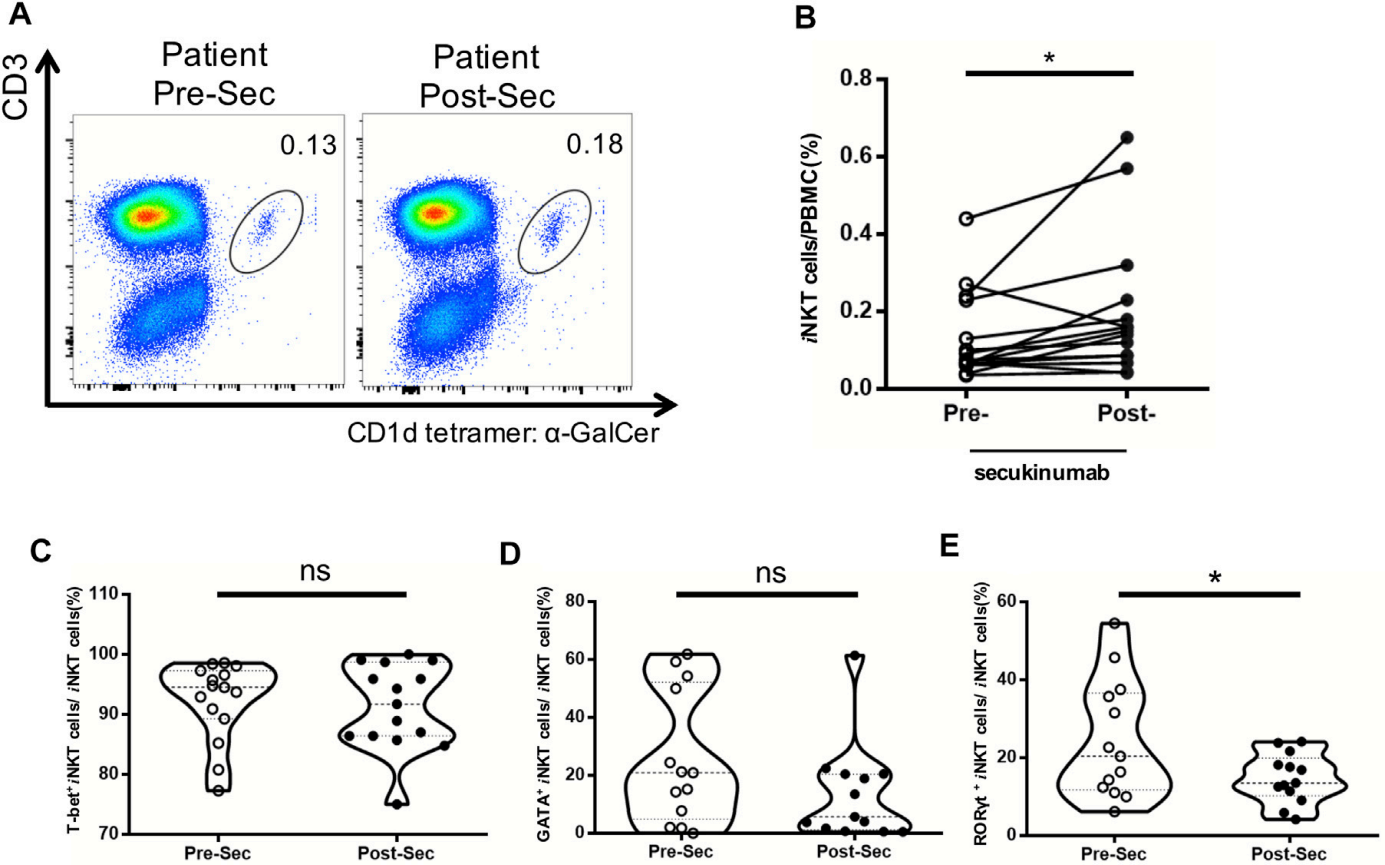
Increased frequency of *i*NKT cells in psoriasis patients treated with secukinumab. PBMC were isolated from psoriasis patients (n = 15) at baseline untreated (Week 0) and 12 Weeks post treatment with secukinumab. **(A)** Representative FACS dot plots for *i*NKT cells from psoriasis patients before and after secukinumab treatment versus healthy controls. **(B)** Summary plots showing individual results of *i*NKT cell frequency in psoriasis patients before and after secukinumab treatment. **(C)** Summary plots showing individual results of T-bet^+^ *i*NKT cells frequency in psoriasis patients before and after secukinumab treatment. **(D)** Summary plots showing individual results of GATA3^+^ *i*NKT cells frequency in psoriasis patients before and after secukinumab treatment. **(E)** Summary plots showing individual results RORγt^+^ *i*NKT cells frequency in psoriasis patients before and after secukinumab treatment. Data show mean +SEM. *p*-values were determined by paired Student’s t-test. ns, no significance, *p < 0.05, **p < 0.01, ***p < 0.001 and ****p < 0.0001.

Secukinumab is effective in the treatment of moderate and severe plaque psoriasis. Patients’ characteristics, PASI, PGA and BSA score before and after the treatment are shown in Table 2. We further analyzed the proportion of *i*NKT cells in the PBMCs of psoriasis patients before and after they treated with secukinumab, and found an increase of *i*NKT cells after the treatment (Figures 5A,B). Moreover, we found a decrease of *i*NKT17 subset in *i*NKT cells after the treatment, while *i*NKT1 and *i*NKT2 subset remained no change (Figures 5C–E).

**TABLE 2:** Details of moderate-to-severe plaque psoriasis patients treated with secukinumab.

Patient characteristics	Baseline (N = 15)	Week 12 (N = 15)
Age, years, mean (SD)	36.53 ± 12.08	36.76 ± 12.08
Female sex, n (%)	26.7%	26.7%
Body mass index	23.7 ± 4.59	23.18 ± 4.18
Disease duration, years, mean (SD)	12.73 ± 5.52	12.96 ± 5.52
PASI score, mean (SD)	18.81 ± 9.78	0.82 ± 0.71
PGA score, mean (SD)	2.27 ± 0.44	0.67 ± 0.47
BSA (%), mean (SD)	33.67 ± 26.19	2.4 ± 2.3
DLQI score, mean (SD)	14.27 ± 5.57	2.93 ± 3.49

## Discussion

In the present study, we isolated mononuclear cells from peripheral blood and detected the expression of *i*NKT cells, conventional T cells and their cytokine production, as well as Treg cells by flow cytometry. Our study represents a large-scale systemic analysis of the basic immunophenotypes of psoriasis patients with strictly matched healthy individual controls. We found that there were no differences between psoriasis patients and the healthy controls with regard to the percentages of conventional CD4^+^ and CD8^+^ T cells, naïve, memory CD4^+^ and CD8^+^ T cells and active CD8^+^ T cells. However, an increase in CD4^+^ T cell activation was found in psoriasis patients. IL-17A is a key cytokine that participates in the pathogenesis of psoriasis. Biologic agents targeting IL-17A or IL-17RA have been demonstrated to have considerable clinical impact in the treatment of psoriasis, and this further proves the vital role of IL-17A in psoriasis (Leonardi et al., 2012; Papp et al., 2012; Van De Kerkhof et al., 2016). Marcel *et al.* showed that the proportion of IL-17A-producing CD8^+^ T cells in the blood of psoriasis patients correlates with their PASI score (Teunissen et al., 2014). In our study, we found that the Th17 and Tc17 populations were significantly increased in the PBMCs of psoriasis patients, in accordance with previous studies (Teunissen et al., 2014; Dainichi et al., 2018). We also found that the population of Tc1 cells was significantly increased in psoriasis patients. However, there were no differences in the percentages of Th1, Th2, and Tc2 cells between psoriasis patients and healthy controls. Treg cells are a subset of T cells that can suppress the inflammation induced by other T cells in autoimmune diseases (Jorn Bovenschen et al., 2011; Barbi et al., 2014; Deng et al., 2016; Ma et al., 2018). Several studies have shown the decreased number and impaired suppressive capacity of Treg cells in autoimmune diseases (Read et al., 2000; Balandina et al., 2005; Zhou et al., 2009; Bailey-Bucktrout et al., 2013; Nakagawa et al., 2016). But research results of Treg cells in peripheral blood of psoriasis varies a lot. Furuhashi found that the number of Treg cells in the PBMCs of severe psoriasis patients with PASI >12 decreased significantly, and the number of Treg cells increased after phototherapy (Fabio et al., 2018). Karamehic’s study showed that the number of CD4^+^CD25^+^Treg cells in the PBMCs of psoriasis patients was significantly lower than that of healthy control, but there was no significant correlation with PASI score (Dainichi et al., 2019). In the meantime, the results of multiple studies have shown that the number of Treg cells in the peripheral blood of psoriasis patients is not significantly different from that of healthy control or is more than healthy control (Dantas et al., 2016; Zhang et al., 2016). In our study, we found that the number of CD4^+^ CD25^+^ FoxP3^+^ Treg cells was also not altered in psoriasis patients, but we did not conduct in-depth research on Treg cells.

As mentioned earlier, in the present study, we used CD1d-tetramer staining, a specific method for *i*NKT identification, to accurately determine the percentage of *i*NKT cells. A significant decrease in peripheral blood *i*NKT cells was observed in psoriasis patients. Interestingly, we found that the proportion of *i*NKT cells increased in patients treated with IL-17A inhibitor secukinumab. Combined with the increased NKT cells in psoriatic lesions reported in previous studies (Bonish et al., 2000; Cameron et al., 2002; Ottaviani et al., 2006; Zhao et al., 2008). We speculated that the *i*NKT cells in the PBMCs have accumulated in psoriatic plaques. And when the lesions subside, *i*NKT cells may come back to peripheral blood.

Human *i*NKT cells can be segregated into CD4^+^ and CD4^−^ subsets according to their phenotypic and functional characteristics (Gumperz et al., 2002). CD4^+^ *i*NKT cells produce both Th1 and Th2 cytokines, whereas the CD4^−^ subset exhibits a Th1 cytokine profile. However, we found no difference in CD4^+^ and CD4^−^ subsets between psoriasis patients and healthy controls. We also examined the sublineages of *i*NKT cells and found that the MFI of GATA3^+^ *i*NKT cells and RORγt^+^ *i*NKT cells were significantly increased in psoriasis patients. We further analyzed the ratio of GATA3^+^ *i*NKT cells vs ROR-γt^+^ *i*NKT cells and found a decrease in psoriasis patients. Besides, the ratio of ROR-γt^+^ *i*NKT cells vs T-bet^+^ *i*NKT cells increased in psoriasis patients, which indicated that *i*NKT cells are more likely to differentiate into ROR-γt^+^ *i*NKT cells in psoriasis. We also found a decrease of *i*NKT17 subset in *i*NKT cells after secukinumab treatment in psoriasis patients. This indicated a potential interaction between IL-17 and *i*NKT cells. Perhaps the decreased proportion of *i*NKT17 cells might be a counterbalance to the administration of IL-17A agonist. We cannot exclude the possibility that the alternation in *i*NKT cells might correlate with disease remission caused by secukinumab treatment. To explore the inner mechanism, we will stimulate *i*NKT cells with IL-17 to examine their interactions in our future research.

Although *i*NKT cells constitute only a small fraction of lymphocytes, their ability to rapidly secrete large amounts of cytokines, make them an important regulator of the Th1, Th2, and Th17 cytokine balance in immune responses. In our study, we found a decrease in the CD69^+^ subset in psoriasis patients. This indicated that psoriasis patients may have less activated *i*NKT cells than healthy controls. But we also found that *i*NKT cells in psoriasis patients secreted higher levels of IL-4 and IL-17, which is consistent with the increase observed in the sublineages of *i*NKT cells in psoriasis patients. In our opinion, the reason why *i*NKT cells in psoriasis patients secreted higher levels of IL-4 and IL-17 mainly lies in the increase of *i*NKT2 and *i*NKT17 subsets. And the inflammatory environment in psoriasis patients may be the reason for *i*NKT functional lineage differentiation shift. Also, the IFN-γ producing *i*NKT1 may express higher CD69 than *i*NKT2 and *i*NKT17. While the proportion of *i*NKT2 and *i*NKT17 augmented in psoriasis patients, the CD69 expression decreased relatively. *i*NKT cells anergy may also be a reason for the decrease of CD69^+^ *i*NKT cell in the PBMCs of psoriasis patients, although it’s not the primary mechanism. It has also been reported that Treg cells suppress NKT cell tumoricidal function by inducing more CD4^−^ NKT cell anergy and less CD4^+^ NKT cell anergy (Ihara et al., 2019). Therefore, there may be interaction between Treg cells and NKT cells in psoriasis, which is also the direction of our further research.

We didn’t found difference in the ratio of IL-17-producing *i*NKT cells vs IFN-γ-producing *i*NKT cells and the ratio of IL-4-producing *i*NKT cells vs IFN-γ-producing *i*NKT cells between psoriasis patients and healthy controls, which indicated there were no imbalance between them. But this result still needs to be verified on more samples.

Based on the increase in Th17 and Tc17 levels, lower proportion and level of activation of *i*NKT cells, increase in the population of *i*NKT17 cells and higher proportion of *i*NKT cells after secukinumab treatment in psoriasis patients, we speculate that dysregulated *i*NKT cells may be involved in the pathogenesis of psoriasis.

## Data Availability

The original contributions presented in the study are included in the article/Supplementary Material, further inquiries can be directed to the corresponding authors.
