# Supplementary material for: Dysregulated Peripheral Invariant Natural Killer T Cells in Plaque Psoriasis Patients
Source: Front Cell Dev Biol. 2022 Feb 3;9:799560. doi: 10.3389/fcell.2021.799560 (PMC8850372; doi:10.3389/fcell.2021.799560)
Supplement: Supplementary file 1 [file DataSheet1.docx]

**Table 1: Details of moderate-to-severe plaque psoriasis patients**

| Characteristics | Healthy Control（N=40） | Psoriasis Patients（N=40） |
| --- | --- | --- |
| Age, years, mean(SD) | 40.1±10.05 | 40.85±9.81 |
| Female sex, n(%) | 45 | 45 |
| Body mass index | 23.87±2.44 | 26.34±4.21 |
| Disease duration, years, mean(SD) | - | 16.63±9.21 |
| PASI score, mean (SD) | - | 18.88±7.63 |
| PGA score, mean (SD) | - | 2.25±0.43 |
| BSA(%) , mean (SD) | - | 39.53±5.59 |
| DLQI score, mean (SD) | - | 11.86±7.17 |

**Table 2: Details of moderate-to-severe plaque psoriasis patients treated with secukinumab**

| Patient characteristics | Baseline（N=15） | Week 12（N=15） |
| --- | --- | --- |
| Age, years, mean(SD) | 36.53±12.08 | 36.76±12.08 |
| Female sex, n(%) | 26.7% | 26.7% |
| Body mass index | 23.7±4.59 | 23.18±4.18 |
| Disease duration, years, mean(SD) | 12.73±5.52 | 12.96±5.52 |
| PASI score, mean (SD) | 18.81±9.78 | 0.82±0.71 |
| PGA score, mean (SD) | 2.27±0.44 | 0.67±0.47 |
| BSA(%) , mean (SD) | 33.67±26.19 | 2.4±2.3 |
| DLQI score, mean (SD) | 14.27±5.57 | 2.93±3.49 |
