# Supplementary material for: Dysregulated Peripheral Invariant Natural Killer T Cells in Plaque Psoriasis Patients
Source: Front Cell Dev Biol. 2022 Feb 3;9:799560. doi: 10.3389/fcell.2021.799560 (PMC8850372; doi:10.3389/fcell.2021.799560)
Supplement: Supplementary file 2 [file Image1.PDF]

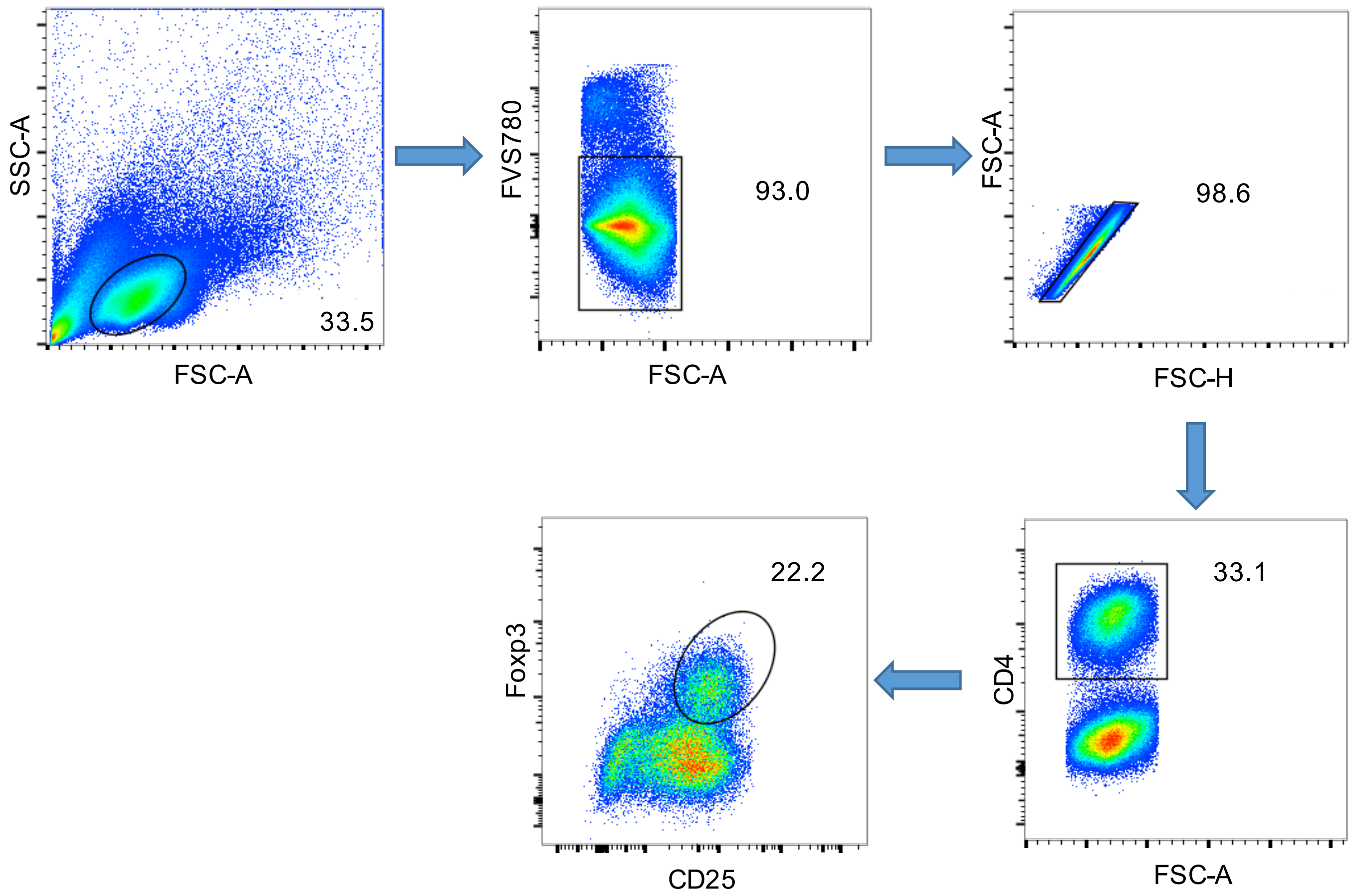

Figure S1. Flow cytometry gating strategy to identify Tregs

PBMC were stained with anti-CD4, CD25, and Foxp3. Total events were gated on the basis of FSC-A vs SSC-A, followed by gating on viable cells. Singlets were gated to exclude doublets from the live lymphocyte population on the basis of FSC-H and FSC-A. $CD4^+$ T cells were gated on the basis of $CD4^+$ and FSC-A. Treg cells were then identified as $Foxp3^+$ and $CD25^+$.

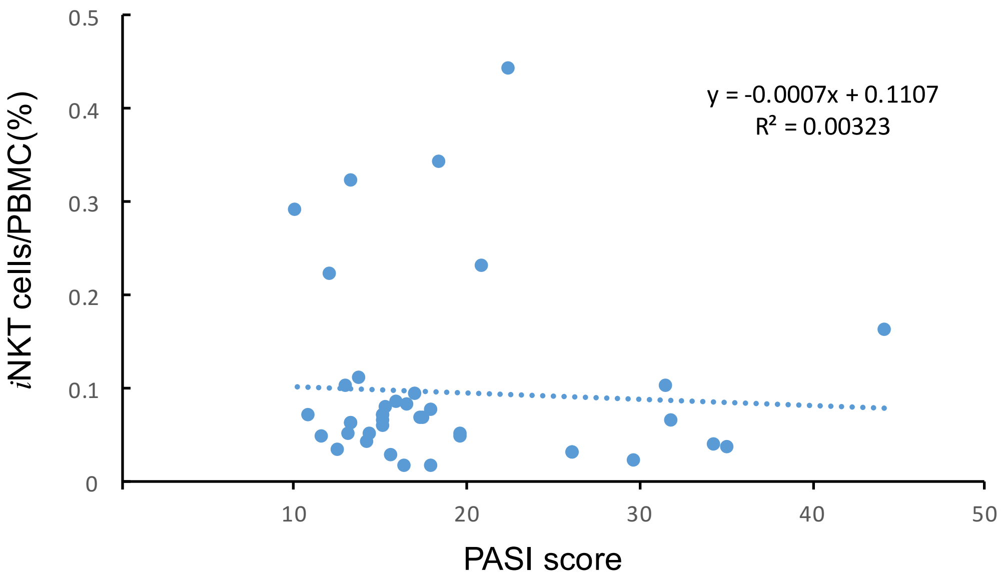

Figure S2. Correlation between *i*NKT cells and PASI score
